# High-Quality Large-Magnification Polymer Lens from Needle Moving Technique and Thermal Assisted Moldless Fabrication Process

**DOI:** 10.1371/journal.pone.0146414

**Published:** 2016-01-14

**Authors:** Ratthasart Amarit, Atcha Kopwitthaya, Prasit Pongsoon, Ungkarn Jarujareet, Kosom Chaitavon, Supanit Porntheeraphat, Sarun Sumriddetchkajorn, Thaweesak Koanantakool

**Affiliations:** 1 National Electronics and Computer Technology Center (NECTEC), Pathum Thani, Thailand; 2 National Science and Technology Development Agency (NSTDA), Pathum Thani, Thailand; Pohang University of Science and Technology, REPUBLIC OF KOREA

## Abstract

The need of mobile microscope is escalating as well as the demand of high quality optical components in low price. We report here a novel needle moving technique to fabricate milli-size lens together with thermal assist moldless method. Our proposed protocol is able to create a high tensile strength structure of the lens and its base which is beneficial for exploiting in convertinga smart phone to be a digital microscope. We observe that no bubble trapped in a lens when this technique is performed which can overcome a challenge problem found in a typical dropping technique. We demonstrate the symmetry, smoothness and micron-scale resolution of the fabricated structure. This proposed technique is promising to serve as high quality control mass production without any expensive equipment required.

## Introduction

Mobile microscopes are crucial for contemporary optical devices which enable a new generation of point-of-care diagnostic tools[1–4], telemedicine[5], or even classroom-learning tool for distant schools[6]. In order to pursue the above goals and allow people to access this technology easily, price of the microscope needs to be affordable. Taking an advantage of omnipresence of mobile phone equipped with digital camera in many countries, the advent of low-cost cell-phone-based microscope directs a powerful tool for image capturing and transmitting to others or even sharing in social networks. Recently, Smith et al. reported a transformation of phone’s integrated lens and image sensor into a microscope using an affordable 1-mm ball lens[7]. Additionally, Skandarajah et al. have constructed a mobile phone microscope structure using a commercially microscope eyepiece and an achromatic objective, showing quantitative imaging with micron-scale spatial resolution under various versions of the iPhone and selected Android phones[8]. Although the design of mobile phone microscope has been proposed, development of a set of optical or mechanical components underneath the system is still needed to simplify the setup and miniaturize the system.

The introduction of elastomeric optics, for example, is of interest due to their flexibility, durability, and cost effectiveness.[9–11]There are various applications such as endoscopes[12], antireflection[13], and gratings[14] based on elastomeric materials. In particular, the demand of small lenses suited for portable devices in the forms of smart phone, tablet, and webcam is growing very rapidly. However, the fabrication process still relies on today complicated molding technology which involves with specially designed and specifically-maintaining-schedule equipment and unavoidable toxic chemicals.[15–18]With these limitations, developing a facile method equipped with few quick steps, manufacture-cost elimination, and environmental friendliness for mass production is critically required.

A low-cost semi-molding technique was reported by Kim et al. to fabricate a high-quality polymeric biconvex lenslets using a wetting behavior at the interface[19]. This technique is able to control size and shape of microlenses. Meanwhile, moldless fabrication is an alternative way to produce affordable optical components. Cox et al. proposed a lens fabrication design using inkjet-printing technology obtaining precise micron-size lens arrays from UV-curing optical epoxies.[20] In order to simplify the process and produce milli-size lenses, Lee et al. utilized the uniform gravitational force to create several transparent polydimethylsiloxane (PDMS; a product of Dow Corning Corp, USA) layers that eventually are shaped as a lens.[21]The focal length is gradually tuned by layering droplets, which depends on sequentially layer deposition and temperature curing in the oven. A most recent and simplified process was reported by Sung et al. using inkjet printing of PDMS droplet and heat accelerated curing *in situ*[22]. The focal length of PDMS lens depends on curing temperature and droplet volume. PDMS mixing needs to be in a vacuum chamber to remove bubbles; however, we found that small bubbles trend to regenerate due to the friction between the mixture and container. These small bubbles can be acceptable in microfluidic chip fabrication because they will finally float to the top during the molding process. In contrast, a bubble in optical devices is critical and can change the incoming-light direction resulting in optical aberration. In particular, rapid curing cannot allow bubbles to leak out[23]. Finally, the bubbles are trapping inside or even on the surface of the lens. The trapping bubbles will decrease not only the image quality, but also the production yield of this technique. Moreover, it is worth noting that the design of polymer lens in practical use needs to take into account. Otherwise direct handling a small lens leads the dirt and oil from finger to accumulate on the surface of the lens that eventually reduces the lens quality.

In this work, we propose a smart design of elastomeric lens with quick and easy fabrication. A lens binds to the flat base with high tensile strength using a needle moving technique. We systematically investigate parameters that affect the lens-base adhesion strength as well as lens magnification. In addition, heat distribution profile is examined to deeply understand the lens formation. Our proposed procedure has ability to provide high magnification comparing up to 170x microscope for single lens use without bubble generation.

## Materials and Methods

### Design and fabrication of lens and its base

**Fig 1:**
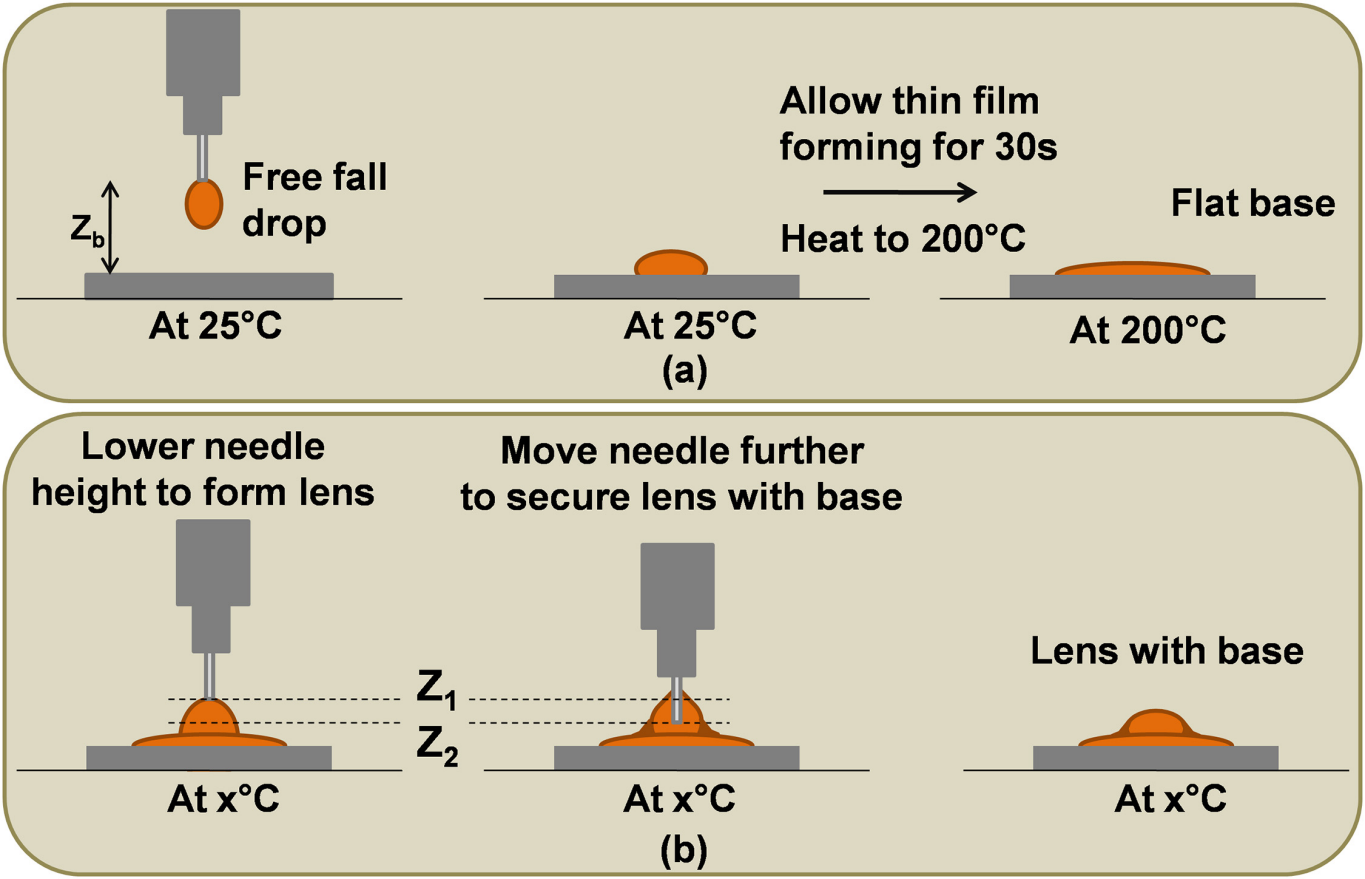
A needle moving technique. Schematic representation of (a) flat base formation using free fall drop of PDMS from a needle with distance Z_b_ away from a glass slide under thermal assisted at 200°C for 20 seconds, and (b) lens fabrication with high tensile strength between lens and its base generated by the proposed moving needle technique.

Liquid solution for making lens and its base is prepared by Sylgard^®^ 184 silicone elastromer kit purchased from Dow Corning, USA. A monomer PDMS is first mixed with a curing agent in the ratio 10:1, and the mixture is placed in the vacuum chamber for 30 minutes to remove air bubbles. At the room temperature, minimal volume of our design machine 2 μl of clear mixture is dropped on a glass slide and allowed to form a flat structure approximately 20 seconds. After that, surface temperature of the glass slide is raised to 200°C to solidify the base. Then, 6–20μl of liquid polymer is dropped on the base with a needle moving technique between height Z_1_ and Z_2_, Fig 1. Finally, the droplet is transformed into plano-convex lens.

### Lens-base tensile strength measurement

**Fig 2:**
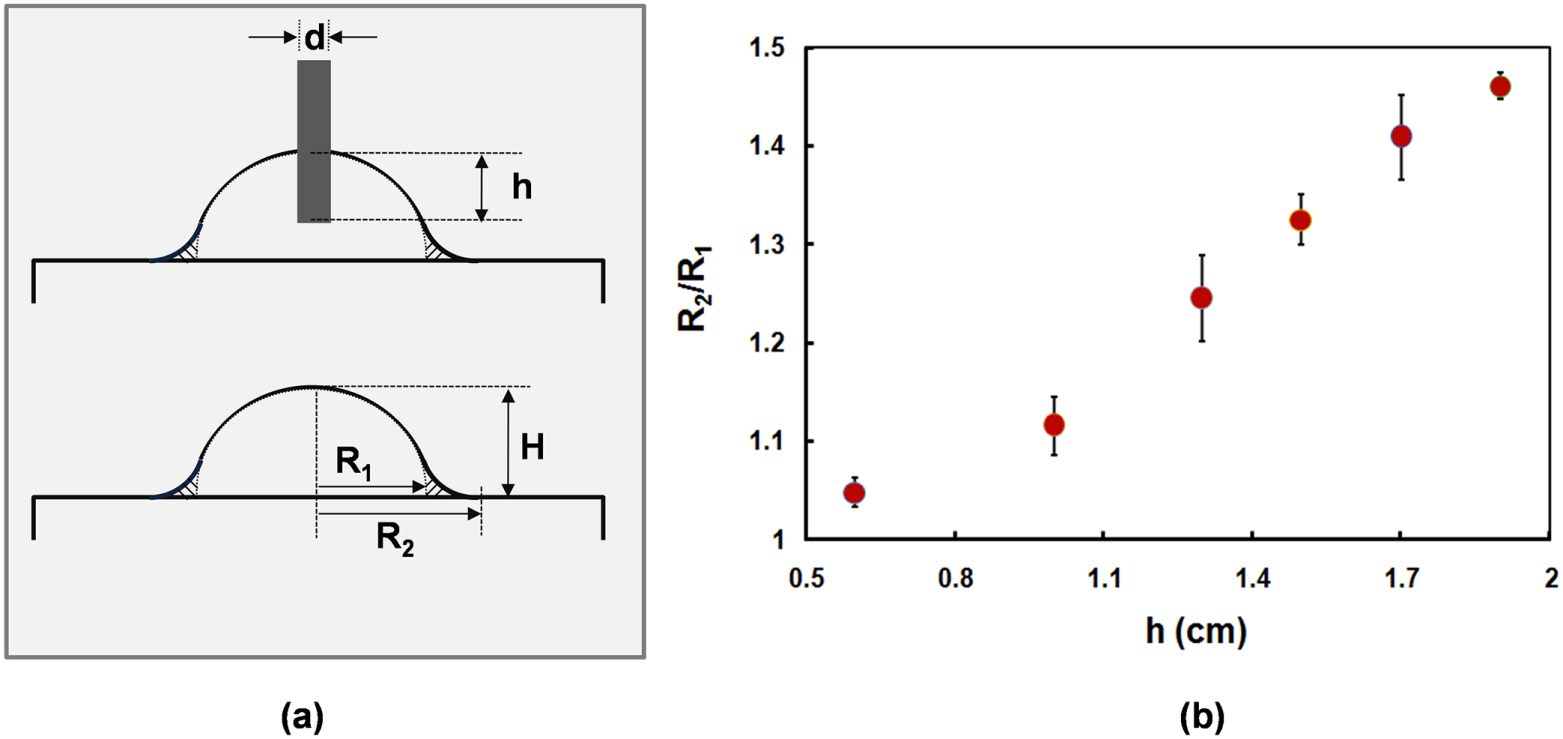
A high tensile lens created by different needle position. (a) The needle moving technique with the depth h inside the polymer droplet creates a lens which has the radius R_2_ and height H, whereas the typical dropping technique gives a lens with smaller radius R_1_. (b) Experimental data shows that the ratio R_2_to R_1_ depends mainly on the depth h, when H, R_1_ and D are controlled parameters.

The tensile strength between lens and its base is measured using the contact angle goniometer, remé-hart Model 500. In detail, the angles of bending base are controlled by slope of acrylic trapezoid placed under testing lens and base, Fig 2. One end of the lens base is clamped onto the trapezoid and another end is laid on the slope and held by the forceps. Then, a breaking point is determined using different angles of trapezoids.

### Heat transfer

An infrared camera is used to monitor heat transfer from hot surface. The FLIR thermovision A40 camera captures the process of polymerization of lens on hot surface. The color scale is then shown by its analytical software.

### Surface profile

Lens symmetry and roughness is examined by Bruker's Dektak^®^ stylus profilers with a resolution in z-axis down to 1A°. Resolution in x- and y-axis are 0.1 mm. The sample is placed in the chamber and lens profile is collected by scanned needle probe.

## Results and Discussion

We introduce here a two-layer structure which comprises of a polymer lens and its base, to be called “MuEye”. The base provides a space for user for ease of handling. This specifically designed feature prevents the lens from any residuals, i.e., oil from skin or dirt on fingers, left on the lens surface due to touching. In addition, a supporting base provides more holding surface area for a lens to be automatically attached under surface tension force to the smart phone camera comparing with the lens alone.

To create a high tensile strength binding between lens and its base, we propose a needle moving technique on the hot surface (Fig 1). Lens geometry obtained from this method will provide more surface area of lens on its base comparing a typical dropping technique. A base is created by free fall drop of PDMS droplet on a smooth surface at room temperature. When the base becomes flat due to gravity force, the temperature is raised to 200°C to solidify a thin polymer sheet. After that, the needle is moved to position Z_1_ and liquid polymer is released at the final volume 2 μl. Then, the needle is gradually moved further to position Z_2_. When the needle reach position Z_2_, it is gradually pulled upward. The droplet shape finally looks like a lens on the flat base. As both lens and its base are made from the same material, the back reflection from the effect of drastic reflective index change is inherently eliminated.

To study the relationship of the needle position and binding efficiency, we first investigate the lens morphology. The lens fabricated with our proposed needle moving technique tends to spread out on a base comparing to a dropping technique (or inkjet printing[22]). We also find out that our proposed method with needle moving speed of 1mm/s can be repeatedly obtained a no-bubble-trapped lens which is the biggest challenge for PDMS lens fabrication. Although PDMS lens preparing with our method is fast curing which usually shows bubble trapping, the optimum height and speed of needle will minimize bubble generation due to the friction between high viscosity liquid and injected needle is limited. As shown in Fig 2(a), the dropping technique provides a lens with the radius R_1_ while a high tensile lens with a longer radius R_2_ due to the additional tail on the side is generated by our proposed needle moving technique. The value R represents ratio of R_2_ and R_1_ and it depends on the depth of needle position inside the liquid polymer (h). It can be obtained by using Eq 1, where D is the diameter of the needle, H is thickness of the lens measuring from the topmost to the base, and R_1_ is the radius of the fabricating lens when h = 0.

$$R\;=\;\sqrt{\frac{D^{2}h}{2R_{1}^{2}H}+1}$$ (1)

It is obvious that a larger value of h leads to a lens with a wider R_2_ while H is a constant parameter (i.e., H = 2 mm) as shown in Fig 2(b). It is worth noting that the needle is not allowed to touch the base in our proposed needle moving technique because the hot surface of the base induces the polymerization process of liquid polymer inside the needle resulting in a clogged injection.

**Fig 3:**
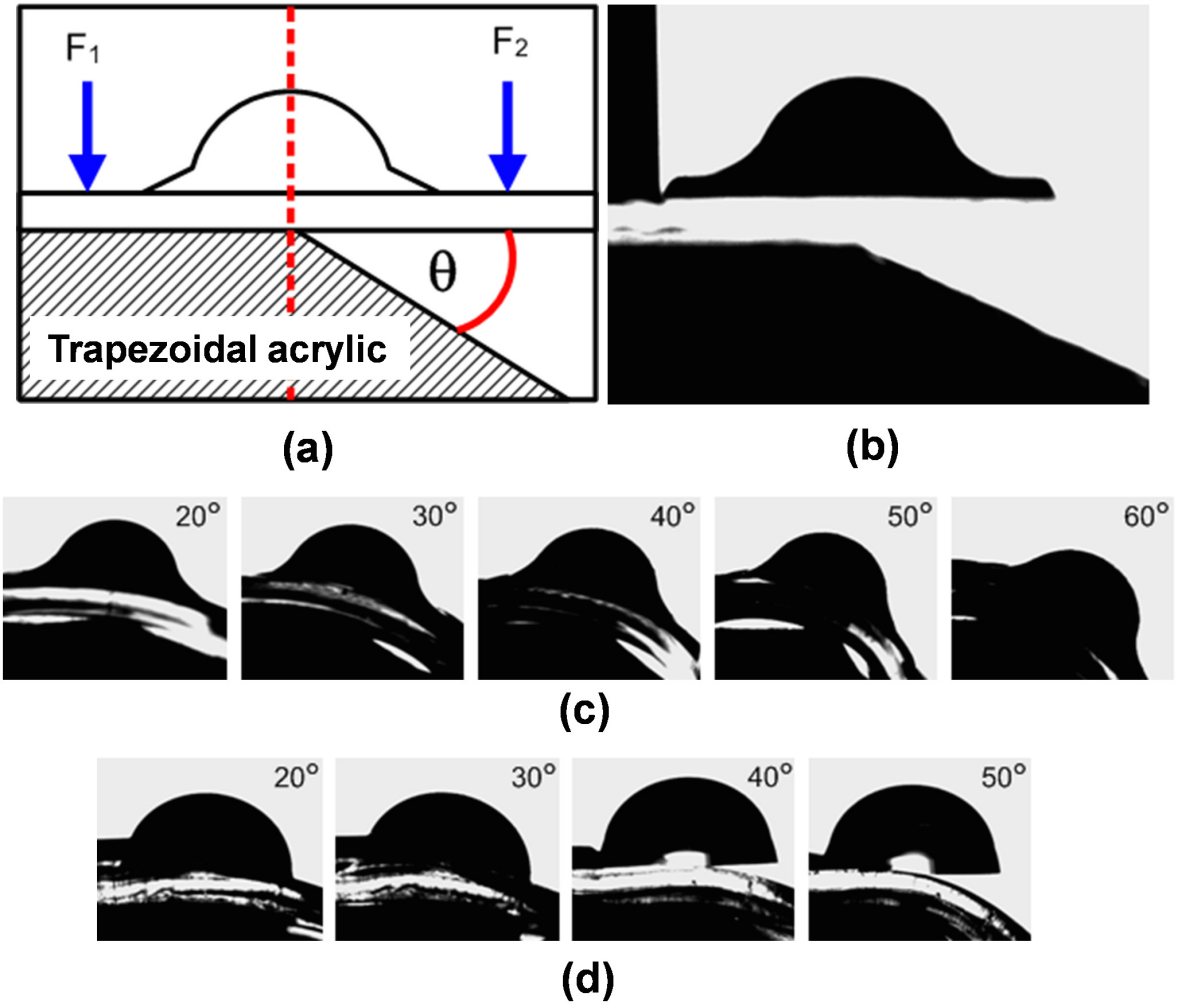
Adhesion strength testing of the lens on the flat base. (a) A schematic of testing method demonstrates an applied force F_2_ and fixed point F_1_ on lens and its base.A piece of trapezoidal acrylic is used to control bending angle. (b) Photo of testing lens and acrylic piece. Contact angle goniometer photos shows (c) a high tensile strength of our proposed needle moving techniques and (d) a breaking point of typical dropping method.

The ratio of R_2_ and R_1_ is also crucial to determine adhesion strength of lens and its base (Fig 3). A strength is investigated using a contact angle goniometer and trapezoidal acrylic with different angle (θ), Fig 3(a,b). The lens created by our proposed needle moving method can resist the tearing up to 60 degree of applying angular force (Fig 3(c)) whereas a typical dropping technique cannot tolerate the applying force. Therefore, it is torn when angular force is applied to less than 40 degree, Fig 3d.

**Fig 4:**
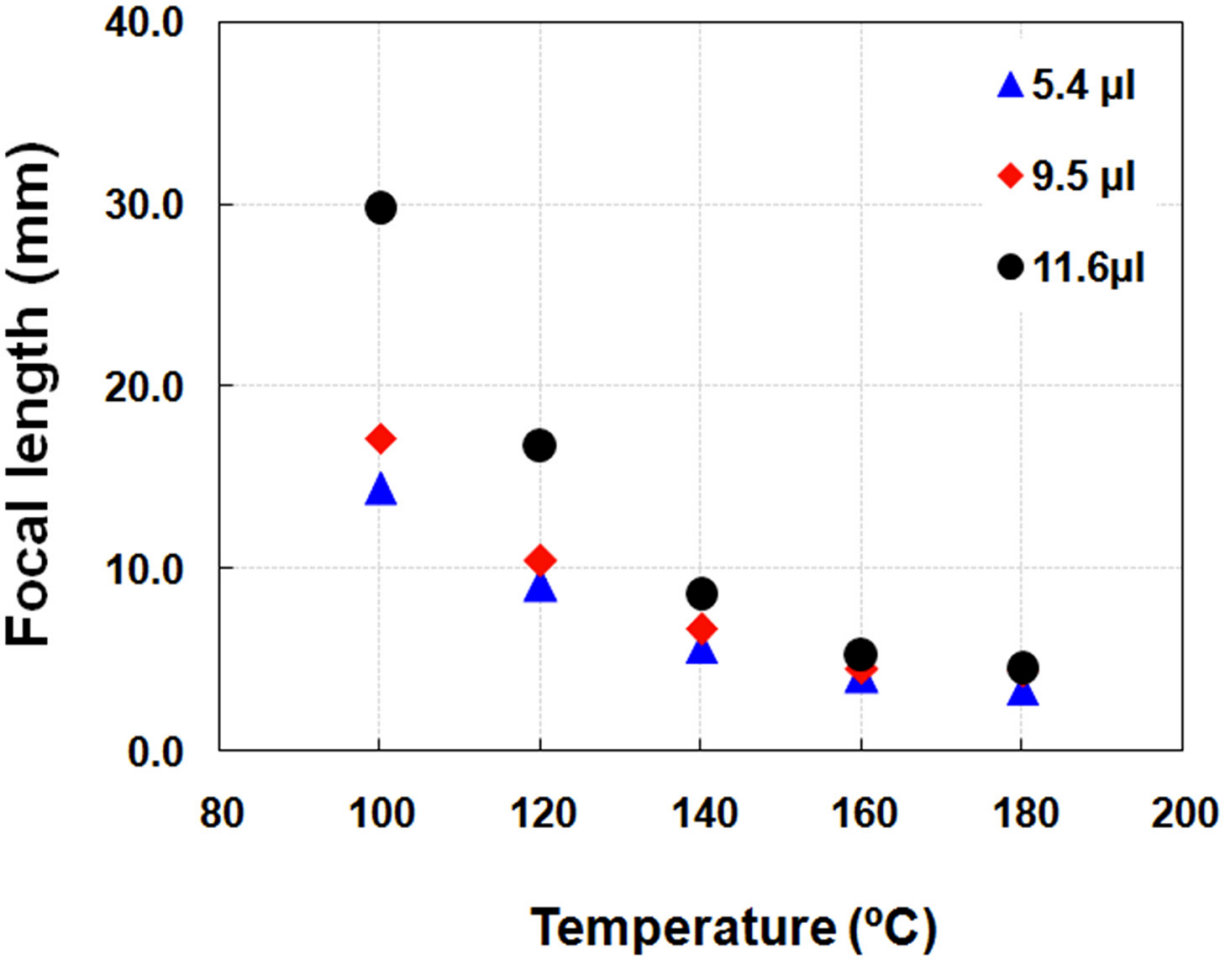
A relationship of lens focal length and fabricated temperature. Measured focal length depends on the surface temperature and the volume of the polymer. Upon temperature increasing, the focal length decreases.

We further study in details of parameters which affect the lens focal length. In this case, the volume of dropping material is varied between 5–12 μl. We find that the lens focal length increases when the volume of droplet increases (Fig 4). Note that the data corresponds to the report of Sung et al[22], although our study mainly investigates in a small volume (<12 μl) while their study shows the volume between 10–200 μl. Taking an advantage of using smaller amount of material, the proposed method can provide a super-low-cost lens. The focal length of the lens is determined by Lensmaker’s equation and radius of curvature is investigated using contact angle goniometer. At surface temperature of 180°C, the focal length of the lens for volume 5.4 μl, 9.5 μl and 11.6 μl are 3.40 ± 0.08, 4.40 ± 0.15, and 4.51 ± 0.01 mm, respectively.

**Fig 5:**
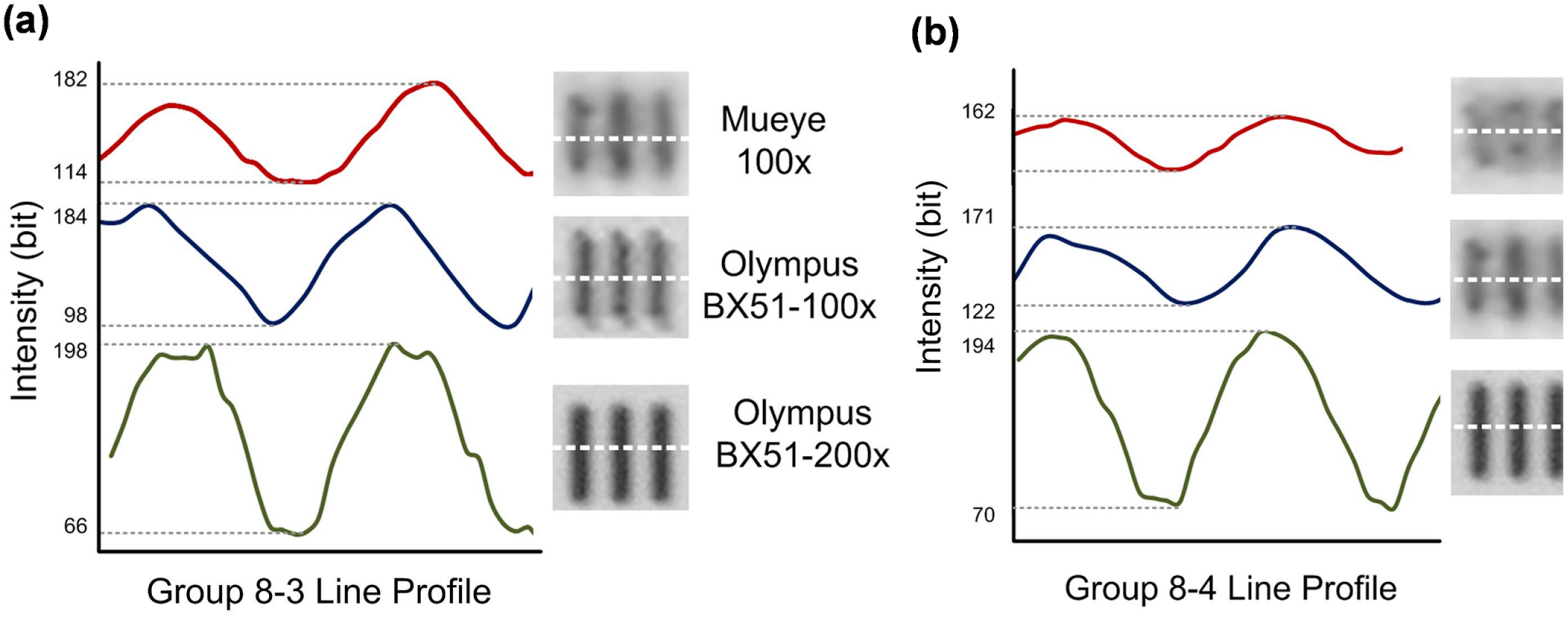
An image quality testing. Line profile plots and images of a standard USAF 1951 pattern: (a) group 8–3 and (b) 8–4. The white dash lines indicate the region of obtaining the intensity data.

The lens quality has also been investigated using a standard USAF 1951 target resolution (#64–862 Edmund optics, Inc). The performance of the system in terms of the image contrast and resolution is determined by comparing with the commercially available Olympus BX51 microscope under the bright field illumination. Fig 5(a) and 5(b) show patterns of group 8 element 3 and group 8 element 4, respectively. Each data is obtained from different system, i.e., red solid plot indicates line profile of the pattern observed by our system (MuEye), blue dash and green dot lines represent line profiles of the pattern under standard objective microscope lenses with 100x and 200x, respectively. This data shows ability of a lens fabricated by our proposed technique to resolve group 8 element 4 with the measured line width of 1.38 μm.

To make the PDMS lens becomes solidified, the heat transfer plays an important role. We therefore study the heat distribution when the droplet is in contact with the hot surface. The continuous heat flux in $\overset{⃑}{z}$-direction ($q_{z}^{"})\;(\frac{W}{m^{2}}$) provides a solid lens according to Fourier’s law of one-dimension heat conduction, as show in Eq 2 where *k* is the thermal conductivity of material $\left(\frac{W}{m}∙K\right),$*T*_*2*_and *T*_*1*_is the temperature at the apex and base of lens respectively, and *L* is thickness of lens[24].

$$q_{z}^{"}\;=\;-k\frac{dT}{dz}\;=\;-k\frac{T_{2}-T_{1}}{L}$$ (2)

**Fig 6:**
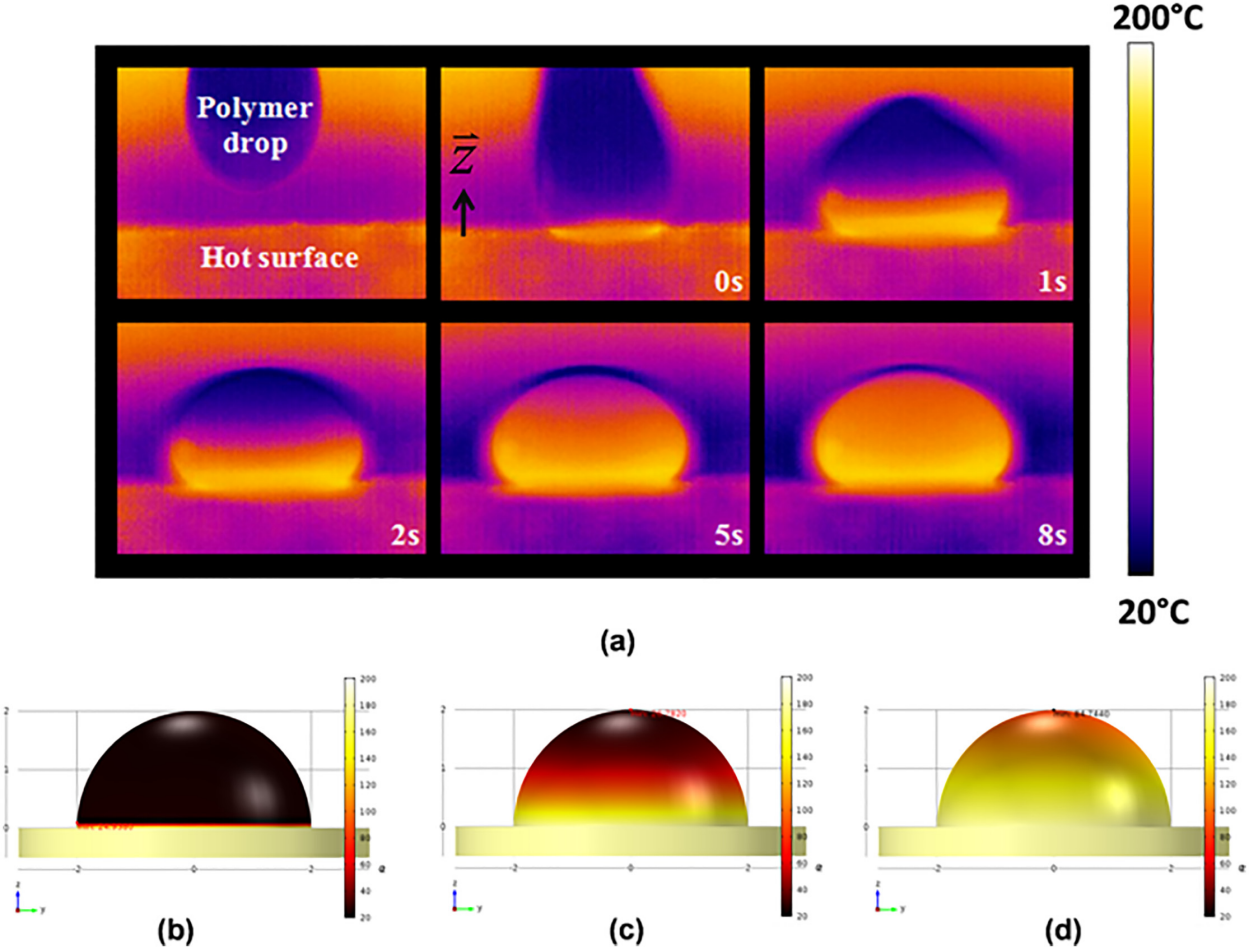
An infrared photo of lens formation. (a) Heat transfer from the hot surface into the polymer during 8 seconds of the lens formation, obtained by heating the surface at 180°C, and 20 μl of polymer droplet. Numerical simulation of heat transfer using shows temperature distribution at (b) 0, (c) 2, and (d) 8 seconds during lens formation.

We observe a rapid heat conduction from the interface between the hot surface and the polymer toward apex of 20 μl of polymer droplet using the FLIR infrared camera in only 8 seconds, on 180°C hot plate (Fig 6). This finding indicates that the lens formation occurs before gravitational force can pull the material off the center, leading to small size and high magnification of lens. When the thickness of material becomes larger, i.e., volume increasing, time of curing becomes longer because more heat flux is required. Then, the Eq 2 can be rewritten as

$$q_{z}^{"}(t)\;=\;-k\frac{T_{2}-T_{1}}{L(v)}$$ (3)

where $q_{z}^{"}(t)$ is heat flux as function of time and *L*(*v*) is thickness of lens depending on droplet volume.

**Fig 7:**
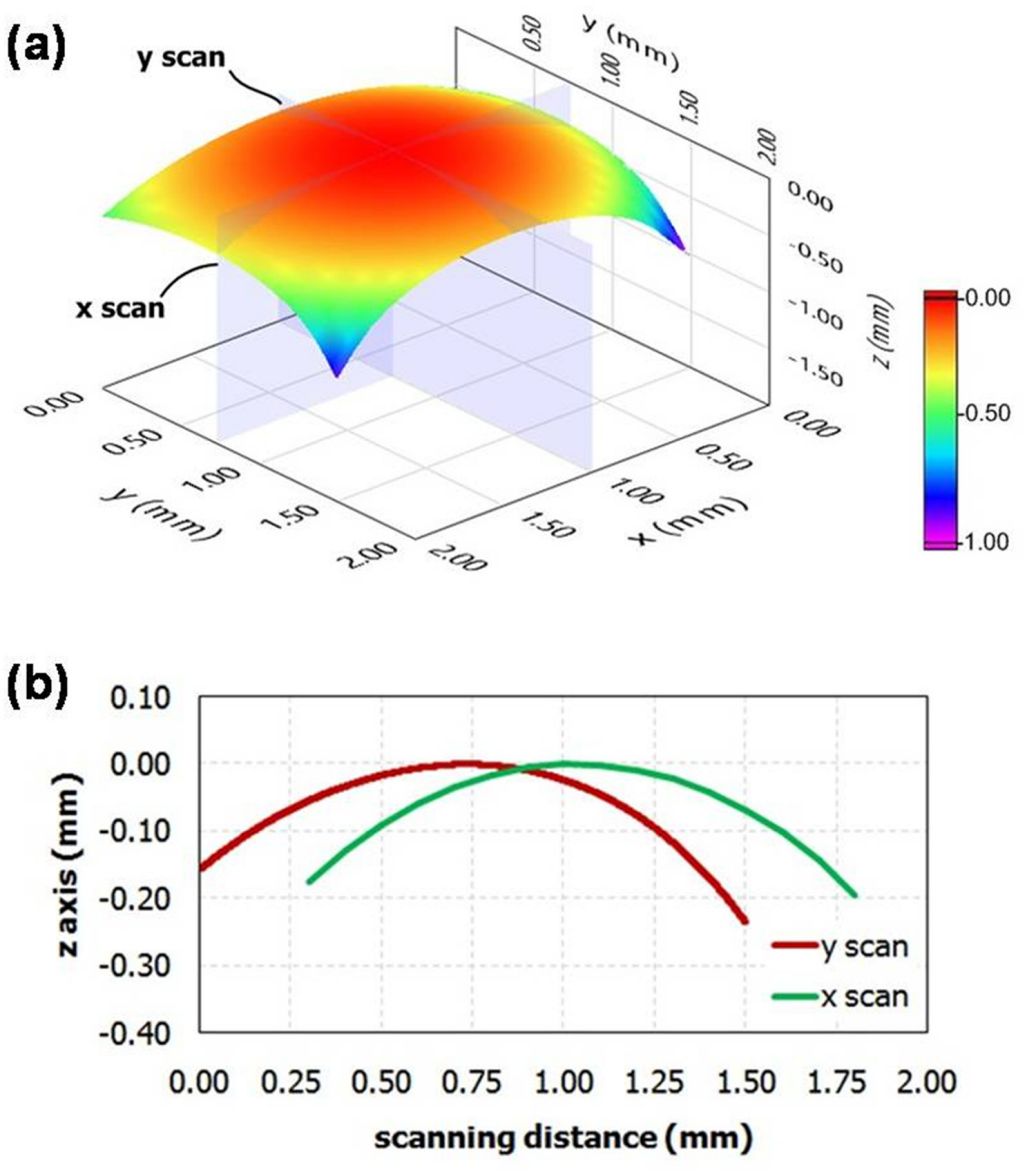
A Lens geometry study. (a) Three-dimensional surface geometry of the fabricating lens, (b) demonstrating symmetry in both x and y axes.

**Fig 8:**
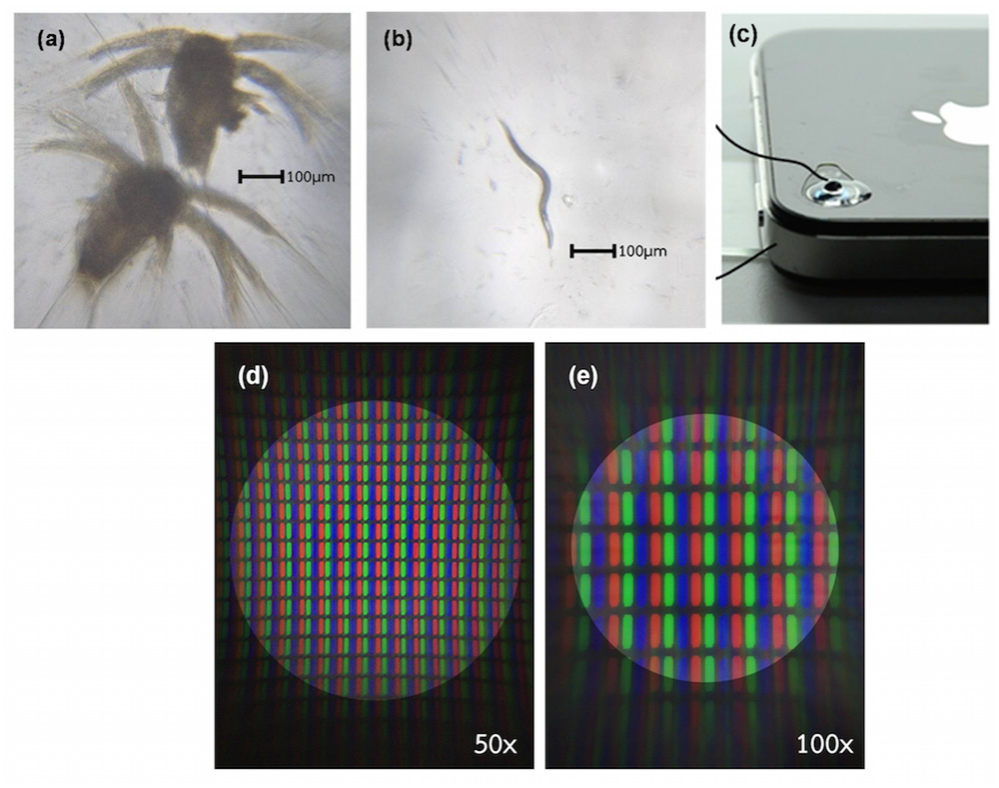
A mobile microscope. Optical photographs of (a) nauplius larvae and (b) aquatic nematodes taken by (c) an iPhone 4S attached with our PDMS lens with its base on the camera. Photographs of RGB pixels demonstrate field of view of (d) 50x and (e) 100x magnification of our preparing lens.

Lens geometry is revealed by Bruker's Dektak^®^ stylus profilers. Fig 7(a) shows 3D plot of lens surface and color scale indicates the position in z- direction whereas Fig 7(b) demonstrates relationship between z-direction and scanning distance in x- and y-direction. It is obvious to conclude that our technique can produce a symmetry and smooth lens. Our high quality lens is attached to a smart phone without any other equipment because of the base property as describe earlier. The images obtained from our system are shown in Fig 8 which is very promising for many application areas such as education, agriculture, healthcare, and food qualification.

## Conclusion

In this paper, we proposed the promising moving needle technique for high quality lens fabrication. The position of needle inside the polymer droplet plays an important role in creating high tensile strength between lens and its base. Our design does not only enable a strong self-adhesive property between base and camera, but also an indirect handling of lens to avoid dirt accumulation. This proposed technique can eliminate air bubble trapped in the polymer lens which is a challenging problem in typical dropping technique. Magnification and focal length of lenses can be tuned by changing fabrication temperature and polymer droplet volume. The lens quality test shows that our polymer lens is comparable to a commercially available microscope with 100x magnification. Therefore, this proposed moving needle technique will be a next step of mass fabrication to produce polymer lenses for mobile microscope. With this opportunity, it can be utilized in many application areas such as education, agriculture, healthcare, and food qualification.
